# Transactions of the American Otological Society

**Published:** 1901-06

**Authors:** 


Transactions of the American Otological Society. Thirty-third annual meeting, Washington, D. C. 1900.

This is not a volume of many pages, but it contains papers of value by some of our most eminent otologists. The one by Dr. Herman Knapp, New York, on Extensive acute caries of the mastoid and petrous portions of the temporal bone, and one by Dr. A. B. Randall, of Philadelphia, on clinical anatomy of the Eustachian tube, are especially notable. Other contributors are Dr. Hiram Woods, Jr., of Baltimore; Dr. E. B. Dench, Dr. Gorham Bacon, Dr. E. Friedenberg, all of New York, and Dr. C. H. Burnett, of Philadelphia. An alphabetical index of otological literature is appended, which is compiled by Dr. H. A. Alderton, of Brooklyn.

